# To attach or not to attach? The effect of carrier surface morphology and topography on attachment of phoretic deutonymphs of *Uropoda orbicularis* (Acari)

**DOI:** 10.1007/s00114-016-1385-9

**Published:** 2016-07-05

**Authors:** Daria Bajerlein, Zbigniew Adamski, Wojciech Kacalak, Katarzyna Tandecka, Maciej Wiesner, Stefan Jurga

**Affiliations:** 1 Department of Animal Taxonomy and Ecology, Faculty of Biology, Adam Mickiewicz University in Poznań, Umultowska 89, 61-614 Poznań, Poland; 2 Electron and Confocal Microscope Laboratory/Department of Animal Physiology and Developmental Biology, Faculty of Biology, Adam Mickiewicz University in Poznań, Umultowska 89, 61-614 Poznań, Poland; 3 Department of Precision Mechanics, Faculty of Mechanical Engineering, Koszalin University of Technology, Racławicka 15-17, 75-620 Koszalin, Poland; 4 Department of Crystal Physics, Faculty of Physics, Adam Mickiewicz University in Poznań, Umultowska 85, 61-614 Poznań, Poland; 5 NanoBioMedical Centre, Adam Mickiewicz University in Poznań, Umultowska 85, 61-614 Poznań, Poland

**Keywords:** *Uropoda orbicularis*, Pedicel, SEM, Profilometry, Attachment devices, Surface roughness

## Abstract

Previous studies on preferences of phoretic deutonymphs of Uropodina for attachment sites have shown that they frequently select smooth and hydrophobic surfaces. The aim of our study was to provide the detailed morphological and topographical characteristics of beetle body surfaces to which deutonymphs frequently attach and to verify how the presence of setae and surface sculpture affects deutonymph attachment. The study was conducted on *Uropoda orbicularis* (Müller, 1776) and its common beetle carriers: *Aphodius prodromus* (Brahm, 1790), *Aphodius fimetarius* (Linnaeus, 1758), *Onthophagus nuchicornis* (Linnaeus, 1758) and *Margarinotus carbonarius* (Hoffmann, 1803). Morphology and topography of elytra, femora, propygidia and pygidia of beetles were analysed mainly using SEM methods supported with CLSM and AFM techniques. The hypothesis that deutonymphs may attach to surfaces covered with setae, if seta density is low enough not to disturb mite movement, was tested. The study revealed that deutonymphs attach to surfaces of various types as follows: (i) smooth, (ii) hairy, i.e., covered with setae, (iii) flat and (iv) sculptured. Smooth body parts and body parts covered with setae of low density were most frequently and intensively occupied with deutonymphs. Surfaces of high seta density were avoided by mites. Within elytra of *Aphodius* beetles, deutonymphs definitely preferred flat surfaces of elytral intervals. On the contrary, densely punctuated propygidium and pygidium in *M. carbonarius* were heavily infested with deutonymphs. We conclude that carrier surface morphology and topography are important for Uropodina deutonymph attachment, but these two factors cannot fully explain the observed relation.

## Introduction

The ability of attachment is a vital feature of living organisms enabling many basic life functions such as nutrition, reproduction and movement. Animals are supplied with various attachment devices that are divided in various ways according to the time of attachment duration, physical mechanism of the system and their biological functions (Gorb 2008; Gorb 2010). A permanent, temporary or transitory attachment are common life strategies in many invertebrates, particularly in parasites, benthic animals and those inhabiting wave-swept shores (Santos et al. 2008; Garner and Litvaitis 2013; Wong and Gorb 2013). The most commonly occurring biological attachment devices are: suckers, hooks, foot pads, claws, spines and clamps (Gorb 2008; Gorb 2010). Moreover, attachment mechanisms may involve wet adhesion through the production of a sticky substance that serve as a biological glue (Gorb 2008; Santos et al. 2008; Van Byern and Grunwald 2010; Li and Zeng 2015).

Various attachment organs have evolved in mites adapted to use other animals to move from one habitat to another, which is called phoresy (Binns 1982; Athias-Binche 1994). In Uropodina mites, the phoretic stage is a deutonymph which developed an ability to secrete a sticky substance that enables a temporary attachment of a mite to its carrier. The substance polymerizes in contact with air and forms a stalk-like structure that is called a pedicel (Faasch 1967). The pedicel is a typical example of a mushroom-shaped attachment structure. It serves as a joint between the mite and its carrier—one terminus of the pedicel adheres to the anal region of the deutonymph and the other adheres to the carrier body surface. After reaching a habitat suitable for further development, the deutonymph leaves the carrier and the pedicel usually remains on the carrier body surface. Up to now, the anatomy and the ultrastructure of the pedicellar gland, morphological diversity of pedicels and carrier-dependent factors affecting pedicel length have been studied (Bajerlein and Witaliński 2012; Bajerlein et al. 2013; Bajerlein and Witaliński 2014). Moreover, we know the sequence of phoretic deutonymph behaviour that leads to pedicel formation (Faasch 1967). Although topical specificity of phoretic deutonymphs of Uropodina is one of the most frequently studied aspects of phoresy in this mite group (Schwarz et al. 1998; Bajerlein and Błoszyk 2004; Bajerlein and Przewoźny 2005; Błoszyk et al. 2006b), the knowledge on morphology and topography of carrier body surfaces to which pedicels attach has never been the subject of more detailed analyses. Many Uropodina species have a wide range of carriers, and it has been shown that phoretic deutonymphs may attach to the same body parts in various beetle species, characterised with different morphology of the body surface (Bajerlein and Błoszyk 2004). Therefore, the aim of our study was to reveal the morphology and topography of attachment surfaces for phoretic deutonymphs of *Uropoda orbicularis*. Moreover, we verified how presence of setae and surface sculpture affects beetle infestation with deutonymphs. We tested the hypothesis that phoretic deutonymphs may attach to surfaces covered with setae if their density is low enough to make deutonymph movement possible. The analysis was conducted on a phoretic mite—*Uropoda orbicularis* (Müller, 1776) and its common beetle carriers from families: Aphodiidae, Scarabaeidae and Histeridae.

## Material and methods

### Study material

The study was conducted on a phoretic mite—*Uropoda orbicularis* (Uropodidae) and its carrier species such as *Aphodius prodromus* (Brahm, 1790) and *Aphodius fimetarius* (Linnaeus, 1758) (Aphodiidae); *Onthophagus nuchicornis* (Linnaeus, 1758) (Scarabaeidae) and *Margarinotus carbonarius* (Hoffmann, 1803) (Histeridae).

**Fig. 1:**
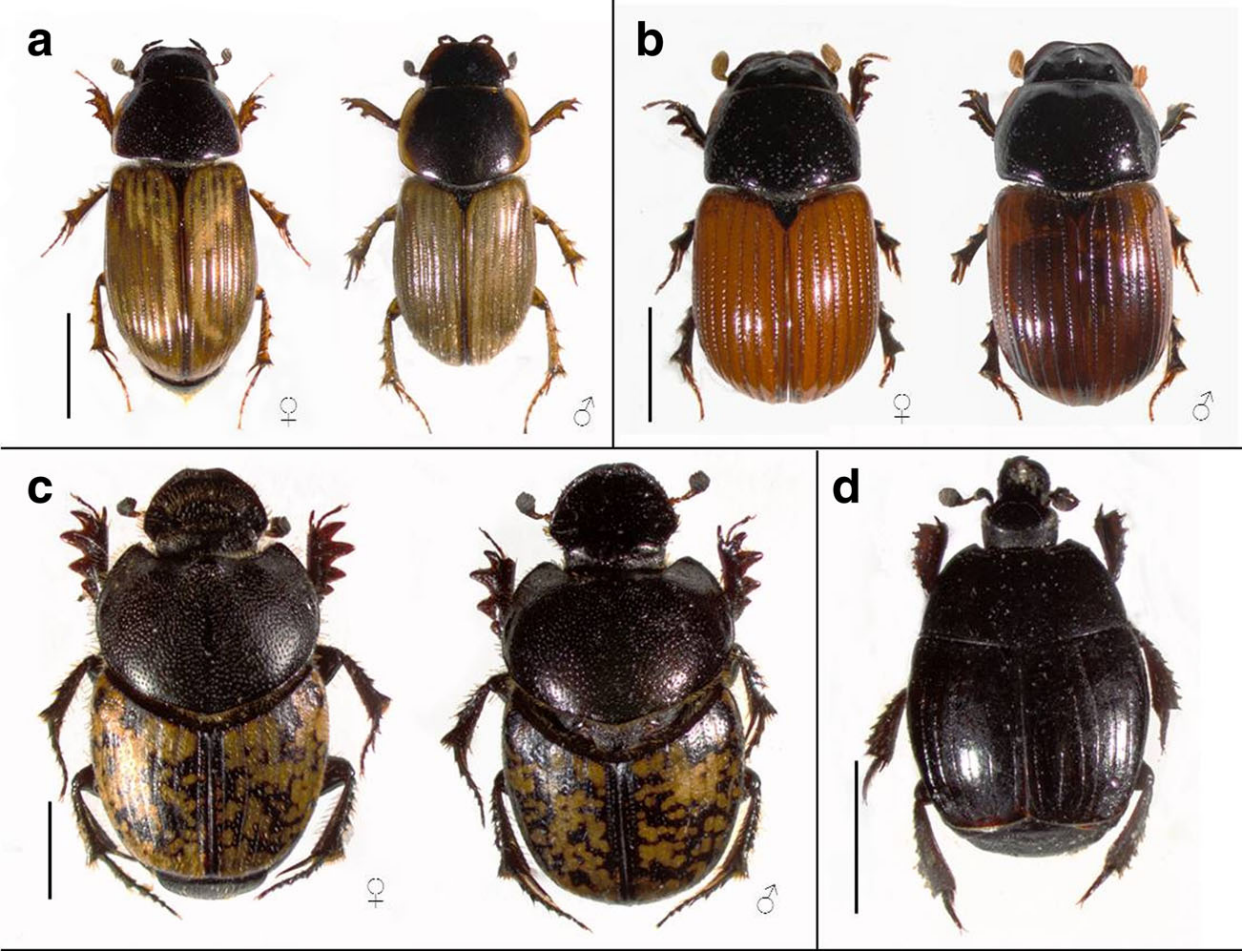
Beetle species studied that serve as carriers of phoretic deutonymphs of *Uropoda orbicularis*. **a** Female and male of *Aphodius prodromus*, **b** female and male of *Aphodius fimetarius*, **c** female and male of *Onthophagus nuchicornis*, **d** *Margarinotus carbonarius. Scale bar* 2 mm

*Uropoda orbicularis* inhabits unstable and patchily distributed microhabitats, e.g., animal manure, compost heaps and bird nests (Faasch 1967; Błoszyk et al. 2006a; Bajerlein 2011). This species is widely distributed in Europe, and its presence was recorded in North America too (Wiśniewski and Hirschmann 1993; Majka et al. 2007). Deutonymphs of *U. orbicularis* have a wide range of beetle carriers including representatives of families as follows: Aphodiidae, Scarabaeidae, Geotrupidae, Histeridae and Hydrophilidae (Mašán 2001; Bajerlein and Błoszyk 2004; Bajerlein and Przewoźny 2005; Bajerlein 2011). *Aphodius prodromus* and *A. fimetarius* are common in temperate regions and in open areas such as pastures (Stebnicka 1976; Hanski and Cambefort 1991). In Poland, they are among the most numerous aphodiid species (Stebnicka 1976). Both males and females of *A. prodromus* have black heads and pronota and yellow elytra with a highly variable and irregular dark stripe pattern (Fig. 1a). Males are usually bigger and their elytra are covered with setae (Stebnicka 1976). Females and males of *A. fimetarius* are characterised with black heads and pronota and red elytra. Representatives of both sexes can be easily distinguished by the presence of an anterior depression on pronotum and three prominent tubercules on the head in males (Stebnicka 1976) (Fig. 1b). *Onthophagus nuchicornis* prefers open habitats characterised by light and dry soil, such as pastures (Stebnicka 1976; Hanski and Cambefort 1991). In the studied beetle community, it was the most numerous representative of the family Scarabaeidae (Bajerlein 2011). The main difference between males and females of *O. nuchicornis* is the presence of a horn on the male’s head and a transverse vertexal ridge on the female’s head. Moreover, females have a small hump on pronotum, behind their head. In both males and females, the heads and pronota are black and the elytra are yellow with an irregular, highly variable, black spot pattern (Stebnicka 1976) (Fig. 1c). *Margarinotus carbonarius* can be frequently found in compost soil, animal manure and under carcasses (Mazur 1981). Typically, to most species from the tribus Histerini, body of this species is black, shiny and oval (Fig. 1d). This species was the most numerous clown beetle in the studied beetle community, and its highest numbers were recorded in May and June (Bajerlein 2011).

Using six dung-baited pitfal traps filled with the ethylene glycol solution, beetles and mites were collected in years 2003–2004 on a pasture grazed by cattle in Wielkopolska region (Western Poland) and emptied at 7-day interval. The specimens were afterwards preserved in 75 % ethylene alcohol solution. To exclude season effect on beetle infestation with mites, only beetles gathered in spring were analysed. Using the key for identification by Stebnicka (1976), beetle species from families Aphodiidae and Scarabaeidae were identified. Beetles from the family Histeridae were identified according to Mazur (1981). Phoretic deutonymphs were mounted on slides and identified using the key for Uropodina identification by Karg (1989) and Mašán (2001).

### Morphometric characteristics of beetle body parts

For each beetle species, we analysed morphology of elytra and femora of the third pair of legs because previous observations on topical specificity in phoretic deutonymphs of *U. orbicularis* have shown that these body parts are most frequently selected for attachment (Bajerlein and Błoszyk 2004).

For the analysis of elytra, 20 individuals of each beetle species were selected. In case of *A. fimetarius*, *A. prodromus* and *O. nuchicornis*, we analysed females (*N* = 10) and males (*N* = 10) separately. For each beetle species/sex, we measured the following: (1) the width and length of elytra, (2) number of intervals and striae, (3) width of the third stria and fourth interval and (4) the length, width and density of elytral setae if present or if their length protrudes above the beetle body surface. Using light stereomicroscopic (SM) digital images, the elytral length and width were measured, whereas using scanning electron microscope (SEM) digital images, the remaining features were measured. The length of elytra was measured as a distance between the base and the apex. The width of elytra was measured as a distance between the outer edges of elytral base. To determine the width of intervals and striae, the fourth interval and third stria were measured. We have chosen these elytral areas because they make the upper surface of each elytron in *A. prodromus*, *A. fimetarius* and *O. nuchicornis*. Since striae have different width along their length, we measured the width in two different locations as follows: in the widest and the narrowest (three measurements for each location) and then the measurements were averaged. Measurements of elytral setae were made for ten randomly selected setae located within the fourth interval on the upper surface of each elytron. Their width was measured within the base of the setae. We counted their number per 100,000 μm^2^ on the upper elytral surface, in order to determine seta density. Moreover, the distance between ten randomly selected elytral setae was measured and then averaged. Some measurements were made specifically for a particular beetle species follow the individual morphology. For *A. prodromus*, we analysed the setae on the upper elytral surface and on the apex of elytra separately. Then, for the statistical analyses, the measurements were averaged. In *O. nuchicornis*, we analysed only the setae on the upper side of elytra because in this species, the elytra are shortened and do not form a distinct slope as in aphodiid species. For elytra in *M. carbonarius*, we measured the width of the third stria and the fourth interval within the area located about 1.3 mm at a distance from the base of elytra since many striae are not present in this area. In clown beetles, the elytra are shortened showing the last two abdominal segments—propygidium and pygidium. Depending on the aim of the study, we analysed these body parts separately or together with elytra, since taking into consideration their location, they correspond to the apex of elytra in *Aphodius* species. For these body parts, their length and width were measured using SM, whereas punctuation density and the diameter of punctures were measured using SEM.

For each beetle species, we analysed morphology of ventral side of 20 femora in randomly selected legs of the third pair (ten in females and ten in males). In case of each leg, using SM digital images, we measured femoral surface area, while using SEM digital images, the length and width of ten randomly selected setae and seta density were measured (number of setae within 100,000 μm^2^).

For the SEM analyses, beetles and pedicels were air-dried, put on a pin stub with double-sided sticky tape, coated with gold and observed in a Zeiss Evo 40 Scanning Electron Microscope (Carl Zeiss SMT Ltd., Cambridge, UK). SM digital images were obtained using an Olympus SZ61 stereomicroscope and Cell A software (Olympus Corporation, Tokyo, Japan).

### Topographic characteristic of elytra, propygidium and pygidium

The surface topography of elytra in all beetle species and propygidium and pygidium in *M. carbonarius* was evaluated using confocal laser scanning microscopy (CLSM). We selected these body parts for the analysis since all of them are located dorsally and have well seen variable surface texture when compared with beetle femora. Surface roughness of the studied body parts was analysed with the LEXT OLS4000 3D Laser Confocal Microscope (Olympus). A measuring field of 705 × 360 μm was scanned in case of each body parts. To obtain the measuring field, 18 scans were taken and then combined. To get the best image quality, all images were taken with ×100 microscope objectives. Surface roughness was presented on profile graphs and 3D surface images. The following 2D parameters were calculated to obtain surface roughness: *Ra*, *Rz* and *Rv* (ISO 4287 2000). *Ra* means arithmetical mean deviation of the assessed profile. *Rz* means maximum height of the assessed profile. *Rv* means maximum profile valley depth of the assessed profile. In addition, we gave values of 3D parameters (*Sa*, *Sz*, *Sv*) (ISO 25178 part 2 2012) which are expanded from 2D parameters. The 3D parameters were calculated for only one surface of given body parts in given species.

### Influence of presence of setae and surface sculpture on deutonymph attachment

To determine the influence of the presence of setae on attachment of deutonymphs of *U. orbicularis*, first, we compared seta length and seta density between beetle species. Then, we determined and compared the prevalence of beetles’ infestation with deutonymphs and deutonymph density on elytra and femora, including beetle sex. Prevalence (*P*) is a proportion of beetles with infested elytra/femora to the total number of all infested beetles and expressed as a percentage. Density (*D*) was calculated as a mean number of deutonymphs on elytra/femora per every beetle individual infested.

To determine deutonymph preferences for attaching to flat or sculptured surfaces, we studied pedicel localization in respect to elytral intervals and striae in randomly selected females and males of *A. fimetarius* and *A. prodromus*. For every sex of the beetle, we analysed 30 individuals. We compared both the frequency of pedicel attachment to intervals and striae and the total number of pedicels attached to the particular elytral area. The first part of the analysis was conducted for *A. fimetarius* and *A. prodromus* separately, whereas the second part was conducted on data pooled from both species. We also analysed the infestation of phoretic deutonymphs on propygidium and pygidium in *Margarinotus carbonarius* since these body parts are characterised with dense punctuation. Deutonymph densities on propygidium, pygidium and elytra as a control surface were compared. The bottom side of the pedicels’ carrier termini attached to surfaces characterised with different texture was observed, documented with SEM and compared with the texture of the beetle cuticle in the contact area. For males of *A. fimetarius*, we evaluated the differences in topography, phase and elastic modulus between elytral intervals and striae with the ICON Bruker Atomic Force Microscope (AFM). Using a high-resolution TESPA-HAR probe, measurements of topography and elastic properties of the abovementioned structures were made with the PeakForce QNM mode. The scanning rate was 0.1 Hz.

### Data analysis

For all morphological features, the mean value, standard error of the mean, minimum and maximum values and the number of measurements were given. Differences between values of morphological features were compared within species and some of them between species. We used one-way ANOVA (if the data follow a normal distribution) and ANOVA Kruskal-Wallis or Mann–Whitney *U* test (if the data did not follow a normal distribution), in order to test the differences between two or more variables. The name of the statistical test used is given in the text and in the figure. For the purpose of multiple pairwise comparisons, Tukey’s HSD test or ANOVA Kruskal-Wallis post hoc test were used accordingly. Although, the ANOVA Kruskal-Wallis is a nonparametric test, we decided to present the mean values, standard error of the mean and standard deviation graphically, instead of median, interquartile range, minimum and maximum, to make the results more readable. Surface roughness parameters were expressed as means ± SD. For prevalence, lower and upper limits were calculated directly from the binomial distribution. To study how the presence of setae affects deutonymph attachment, a correlation coefficient (*r*) between seta and deutonymph densities was calculated.

## Results

### Morphometric analysis of beetle body parts that serve as attachment surfaces for deutonymphs

The detailed morphometric characteristics of studied features are presented in Tables 1, 2 and 3. More general descriptions of morphology of the studied surfaces, including their comparison, are given below.

**Table 1:** Morphometric characteristics of elytra and femora of the third pair of legs in studied beetles

	*Aphodius prodromus* males	*Aphodius prodromus* females	*Aphodius fimetarius* males	*Aphodius fimetarius* females	*Onthophagus nuchicornis* males	*Onthophagus nuchicornis* females	*Margarinotus carbonarius*
	Mean ± SE	Mean ± SE	Mean ± SE	Mean ± SE	Mean ± SE	Mean ± SE	Mean ± SE
	Min–max (*N*)	Min–max (*N*)	Min–max (*N*)	Min–max (*N*)	Min–max (*N*)	Min–max (*N*)	Min–max (*N*)
ElyLen (mm)	3.9 ± 0.04	3.8 ± 0.05	3.8 ± 0.05	3.9 ± 0.06	2.8 ± 0.03	2.9 ± 0.03	2.2 ± 0.04
	3.4–4.4 (30)	3.1–4.2 (30)	3.4–4.3 (30)	3.0–4.3 (30)	2.5–3.1 (30)	2.6–3.3 (30)	(1.8–2.6) (30)
ElyWid (mm)	2.5 ± 0.03	2.4 ± 0.03	3.0 ± 0.04	2.9 ± 0.04	4.0 ± 0.03	4.0 ± 0.04	2.6 ± 0.04
	2.2–2.9 (30)	2.0–2.8 (30)	2.6–3.5 (30)	2.4–3.3 (30)	3.7–4.3 (30)	3.4–4.5 (30)	(2.3–3.1) (30)
ElySetLen* (μm)	68.1 ± 1.3	–	–	–	58.5 ± 1.4	54.7 ± 0.9	–
	17.7–107.4 (202)				18.3–92.9 (188)	18.4–87.3 (189)	
ElySetWid* (μm)	5.2 ± 0.1	–	–	–	7.7 ± 0.1	7.1 ± 0.1	–
	2.8–7.3 (197)				4.2–11.6 (198)	4.3–10.9 (189)	
ElySetDen* (No/10^5^ μm^2^)	19.8 ± 0.6	–	–	–	4.7 ± 0.2	4.6 ± 0.1	–
	11.8–26.2 (33)				2.8–8.0 (30)	3.3–5.8 (32)	
ElySetDis* (μm)	100.5 ± 3.2	–	–	–	136.3 ± 2.4	139.8 ± 2.0	–
	35.4–166.3 (116)				73.1–237.6 (192)	86.2–216.0 (186)	
StrWid (μm)	30.8 ± 0.7	27.3 ± 0.7	45.6 ± 1.1	42.8 ± 1.1	39.2 ± 1.0	40.1 ± 0.9	21.0 ± 0.7
	17.5–48.4 (111)	16.2–44 (119)	24.4–72.2 (120)	23.0–63.9 (120)	18.5–62.4 (116)	18.8–59.2 (114)	8.8–38.0 (109)
IntWid (μm)	158.1 ± 1.6	165.6 ± 2.0	242.5 ± 2.3	243.1 ± 2.6	316.2 ± 5.9	309.9 ± 3.8	202.9 ± 3.4
	126.7–187.7 (81)	129.5–197.7 (80)	196.8–294.5 (80)	201.3–288.8 (80)	233.6–430.6 (81)	242.6–382.6 (76)	133.7–285.6 (90)
ElySetLen^ (μm)	62.0 ± 1.2	27.7 ± 0.6	–	–	–	–	–
	28.9–98.6 (140)	12.7–50.7 (176)					
ElySetWid^ (μm)	6.3 ± 0.1	4.2 ± 0.1	–	–	–	–	–
	3.5–9.1 (110)	1.5–7.2 (186)					
ElySetDen^ (No/10^5^ μm^2^)	34.3 ± 1.1	32.1 ± 0.9	–	–	–	–	–
	22.0–48.7 (31)	25.0–38.0 (16)					
FemArea (μm^2^)	7.8(±0.3) × 10^5^	5.9(±0.1) × 10^5^	7.0(±0.2) × 10^5^	6.6(±0.2) × 10^5^	1.3(±0.05) × 10^6^	1.1(±0.05) × 10^6^	5.0(±0.2) × 10^5^
	(4.8–9.4) × 10^5^ (20)	(4.7–7.4) × 10^5^ (20)	(5.1–8.4) × 10^5^ (20)	(5.2–7.7) × 10^5^ (20)	(1.0–1.7) × 10^6^ (22)	(0.6–1.5) × 10^6^ (21)	(3.4–5.7) × 10^5^ (22)
FemSetLen (μm)	74.3 ± 4.1	99.1 ± 7.5	33.2 ± 4.5	46.7 ± 7.9	128.5 ± 3.4	98.6 ± 2.3	–
	22.5–160.8 (60)	40.7–193.3 (30)	3.8–113.6 (55)	3.0–138.3 (27)	69.1–201.1 (90)	38.3–133.9 (80)	
FemSetWid (μm)	7.9 ± 0.3	9.0 ± 0.5	6.4 ± 0.4	6.2 ± 0.4	11.0 ± 0.3	8.2 ± 0.2	–
	3.5–13.5 (56)	4.6–13.6 (34)	2.4–12.3 (47)	2.6–10.2 (35)	5.8–16.5 (85)	4.3–11.2 (78)	
FemSetDen (No/10^5^ μm^2^)	–	–	–	–	3.2 ± 0.1	3.5 ± 0.3	–
					2.4–4.3 (22)	2.4–6.1 (21)	
FemSetDis (μm)	–	–	–	–	198.3 ± 7.5	162.0 ± 6.7	–
					86.3–402.7 (88)	79.9–317.5 (80)	

*ElyLen* length of elytra, *ElyWid* width of elytra, ElySetLen* length of setae located on upper side of elytra, ElySetWid* width of setae on upper side of elytra, ElySetDen* density of setae on upper side of elytra, ElySetDis* distance between elytral setae on the upper side of elytra, *StrWid* width of elytral striae, *IntWid* width of elytral intervals, *ElySetLen*^ length of setae located on the apex of elytra, *ElySetWid*^ width of setae on the apex of elytra, *ElySetDen*^ density of setae on the apex of elytra, *FemArea* femoral surface area, *FemSetLen* length of femoral setae, *FemSetWid* width of femoral setae, *FemSetDen* density of femoral setae, *FemSetDis* distance between femoral setae, *No* number of setae, “–” lack of data

**Table 2:** Morphometric characteristics of propygidium and pygidium in *Margarinotus carbonarius*

*Margarinotus carbonarius*	ProLen (mm)	ProWid (mm)	ProPunDia (μm)	ProPunDen (No/10^5^ μm^2^)	PygLen (mm)	PygWid (mm)	PygPunDia (μm)	PygPunDen (No/10^5^ μm^2^)
Mean ± SE	0.7 ± 0.01	1.9 ± 0.03	30.2 ± 0.5	22.4 ± 0.7	0.9 ± 0.02	1.4 ± 0.02	23.6 ± 0.5	37.6 ± 1.1
Min–max	0.6–0.9	1.6–2.3	13.2–45.7	17–29	0.7–1.1	1.3–1.7	10.5–36.4	26–48
(*N*)	(30)	(30)	(150)	(22)	(30)	(30)	(122)	(22)

*ProLen* length of propygidium, *ProWid* width of propygidium, *ProPunDia* diameter of punctures on propygidium, *ProPunDen* punctuation density on propygidium, *PygLen* length of pygidium, *PygWid* width of pygidium, *PygPunDia* diameter of punctures on pygidium, *PygPunDen* punctuation density on pygidium, *N* number of measurements, *No* number of punctures

**Table 3:** Types of attachment surfaces (beetle body parts) for deutonymphs of *Uropoda orbicularis* characterised according to the presence of setae and sculpture

	Elytral striae	Elytral intervals	Femora	Propygidium, pygidium
*A. prodromus* females	Smooth, sculptured	Smooth, flat	Smooth, flat	–
*A. prodromus* males	Smooth, sculptured	Hairy, flat	Smooth, flat	–
*A. fimetarius* females	Smooth, sculptured	Smooth, flat	Smooth, flat	–
*A. fimetarius* males	Smooth, sculptured	Smooth, flat	Smooth, flat	–
*O. nuchicornis* females	Smooth, sculptured	Hairy, flat	Hairy, flat	–
*O. nuchicornis* males	Smooth, sculptured	Hairy, flat	Hairy, flat	–
*M. carbonarius*	Smooth, sculptured	Smooth, flat	Smooth, flat	Smooth, sculptured

The criterion of differentiation between smooth and hairy surfaces was both the presence and length of the setae. The surface was characterised as smooth if the setae were not present or if they were so short that they did not protrude above the carrier body surface. The criterion of differentiation between flat and sculptured surfaces was the size of surface irregularities. The surface was characterised as sculptured if the irregularities were distinct and not seen only at microscale

#### Characteristic of elytra in studied beetle species

**Fig. 2:**
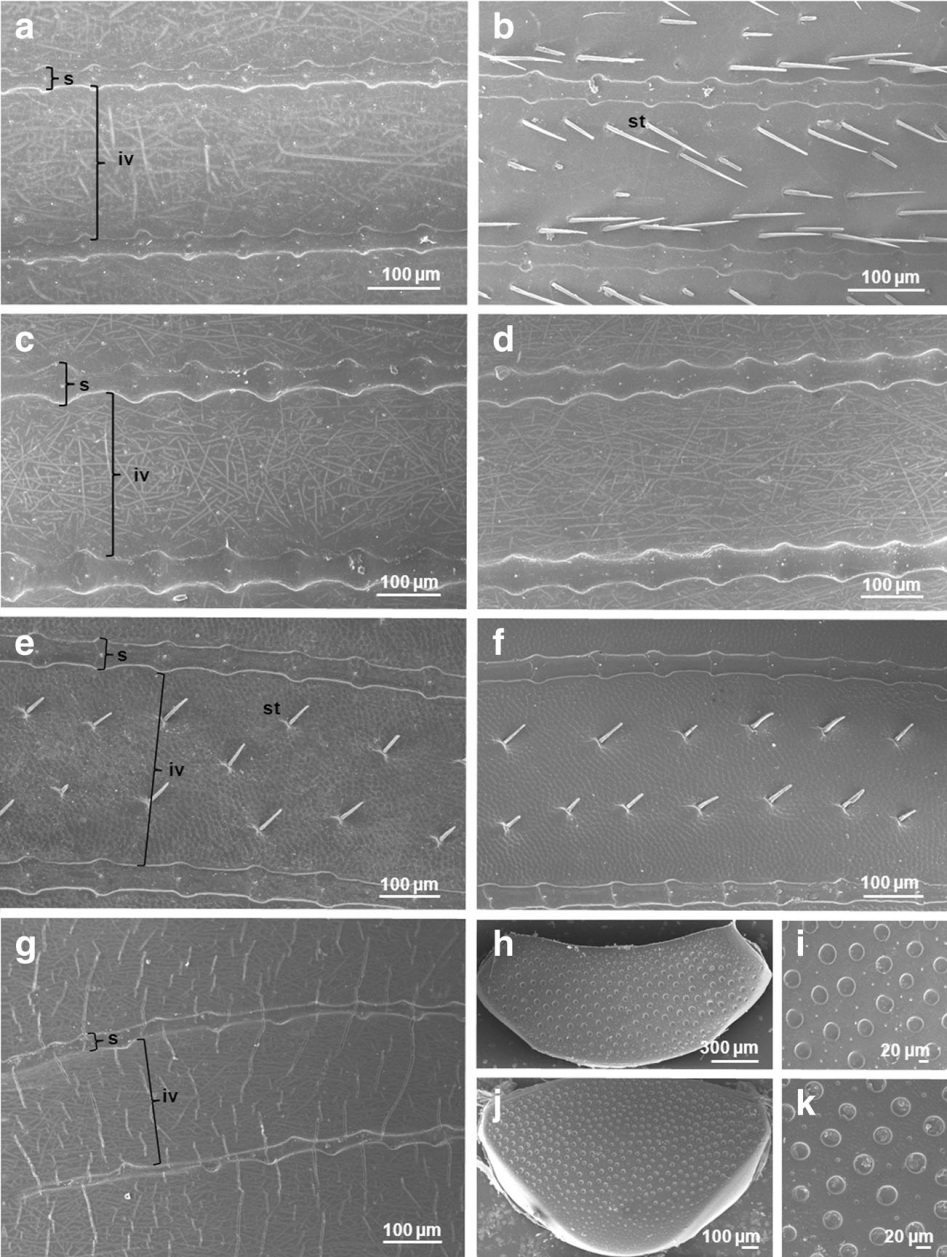
SEM images of the upper surface of elytra in females (**a**) and males (**b**) of *Aphodius prodromus*, females (**c**) and males (**d**) of *Aphodius fimetarius*, females (**e**) and males (**f**) of *Onthophagus nuchicornis*, **g** *Margarinotus carbonarius*, and propygidium (**h**, **i**) and pygidium (**j**, **k**) in *M. carbonarius. iv* interval, *s* stria, *st* seta

Males of *A. prodromus* have bigger elytra than females (width × length, ANOVA, *F*_(1,58)_ = 5.892, *P* = 0.018) (Table 1). In both sexes, the elytra consist of ten intervals and nine punctuated striae. The mean width of the fourth interval in females is 165.6 ± 2.0 μm (mean ± SE) and 158.1 ± 1.6 μm in males (ANOVA, *F*_(1,151)_ = 3.406, *P* = 0.067). In females, the mean width of striae is 27.3 ± 0.7 and 30.8 ± 0.7 μm in males (ANOVA, *F*_(1,228)_ = 11.688, *P* = 0.0008). Both in females and males, the widened parts of the striae bear single setae in their central area. The setae are very short and do not protrude above the height of the striae. The main difference in elytral morphology between females and males is the presence of distinct setae on intervals in males (Fig. 2a, b). The small area of elytral base in males bears very short setae, whereas the remaining part of elytra is covered with a densely distributed setae (26.7 ± 1.1 setae/100,000 μm^2^), with a mean density of 19.8 ± 0.6 setae/100,000 μm^2^ on the upper part of elytra and 34.3 ± 1.1 setae/100,000 μm^2^ on the apex. Setae located within the upper part of elytra are arranged in two main rows along both sides of an interval with some setae located in the central part of the interval. Setae located on the upper part of elytra have the mean length of 68.1 ± 1.3 μm and width of 5.2 ± 0.1 μm, whereas those situated on the apex are shorter (62.0 ± 1.2 μm) and have a width of 6.3 ± 0.1 μm. In females, elytral intervals bear very short setae, which are arranged in two rows that can be seen only at high magnification. Thus, it could be stated that almost the entire elytral surface is smooth in females, with an exception of the apex which is covered with longer setae (27.7 ± 0.6 μm) of a mean density of 32.1 ± 0.9 setae/100,000 μm^2^.

Both sexes of *A. fimetarius* have elytra of a similar size (ANOVA, *F*_(1,58)_ = 0.022, *P* = 0.883), which consist of ten intervals and nine punctuated striae. The mean width of intervals in females is 243.1 ± 2.6 μm and 242.5 ± 2.3 μm in males (ANOVA, *F*_(1,158)_ = 0.029, *P* = 0.866). Striae in females have a mean width of 42.8 ± 1.1 and 45.6 ± 1.1 μm in males (ANOVA, *F*_(1,238)_ = 3.300, *P* = 0.071) (Table 1). In both, females and males, the main part of elytra is smooth (Fig. 2c, d). Both in females and males, the widened parts of the striae bear single setae in their central area. Similar to *A. prodromus*, the setae are very short and do not exceed the height of the striae.

Elytra in females and males of *O. nuchicornis* are of similar size (ANOVA, *F*_(1,58)_ = 0.199, *P* = 0.657) and consist of eight intervals and seven striae. Intervals in females have a mean width of 309.9 ± 3.8 and 316.2 ± 5.9 μm in males (ANOVA, *F*_(1,155)_ = 0.774, *P* = 0.380) (Table 1). The entire elytral surface within intervals bears setae, which are longer in males (58.5 ± 1.4 μm) than in females (54.7 ± 0.9 μm) (ANOVA, *F*_(1,377)_ = 7.000, *P* = 0.009) (Fig. 2e, f). Differences in their densities between females (4.6 ± 0.1 setae/100,000 μm^2^) and males (4.7 ± 0.2 setae/100,000 μm^2^) were not statistically significant (ANOVA, *F*_(1,60)_ = 0.372, *P* = 0.544). In both sexes of *O. nuchicornis*, the surface of intervals is covered with irregular polygons. The mean width of striae in females (40.1 ± 0.9 μm) and males (39.2 ± 1.0 μm) is of similar size (ANOVA, *F*_(1,228)_ = 0.505, *P* = 0.478) (Table 1). Just like in the previously characterised beetle species, both females and males of *O. nuchicornis* have elytra with striae bearing very short, single setae in their central area.

The elytra in *M. carbonarius* are smooth, strongly shortened and show propygidium and pygidium, i.e., the last two abdominal segments (Fig. 1d). Seven parallelly situated striae of different lengths and irregular widths and one short, located diagonally near the humeral callus, are present on the elytral surface. Three inner elytral striae are shortened and can be seen only within the latter part of elytra. The remaining four are not shortened. The mean width of the third elytral striae is 21.0 ± 0.7 μm, and the mean interval width is 202.9 ± 3.4 μm (Table 1) (Fig. 2g). The elytral surface within intervals is covered with very short setae that can be seen only at high magnification. Propygidium in *M. carbonarius* is trapezoid, whereas pygidium is triangular (Fig. 2h–k). The main part of both segments is covered with large punctures of a mean diameter of 30.2 ± 0.5 μm for propygidium and 23.6 ± 0.5 μm for pygidium (Table 2). The mean density of large punctuation on propygidium is lower (22.4 ± 0.7 punctures/100,000 μm^2^) than on pygidium (37.6 ± 1.1 punctures/100,000 μm^2^) (ANOVA, *F*_(1,42)_ = 131.86, *P* < 0.00001) (Table 2). Between large punctures, smaller punctures are present (Fig. 2i, k).

#### Characteristic of beetle femora of the third pair of legs

**Fig. 3:**
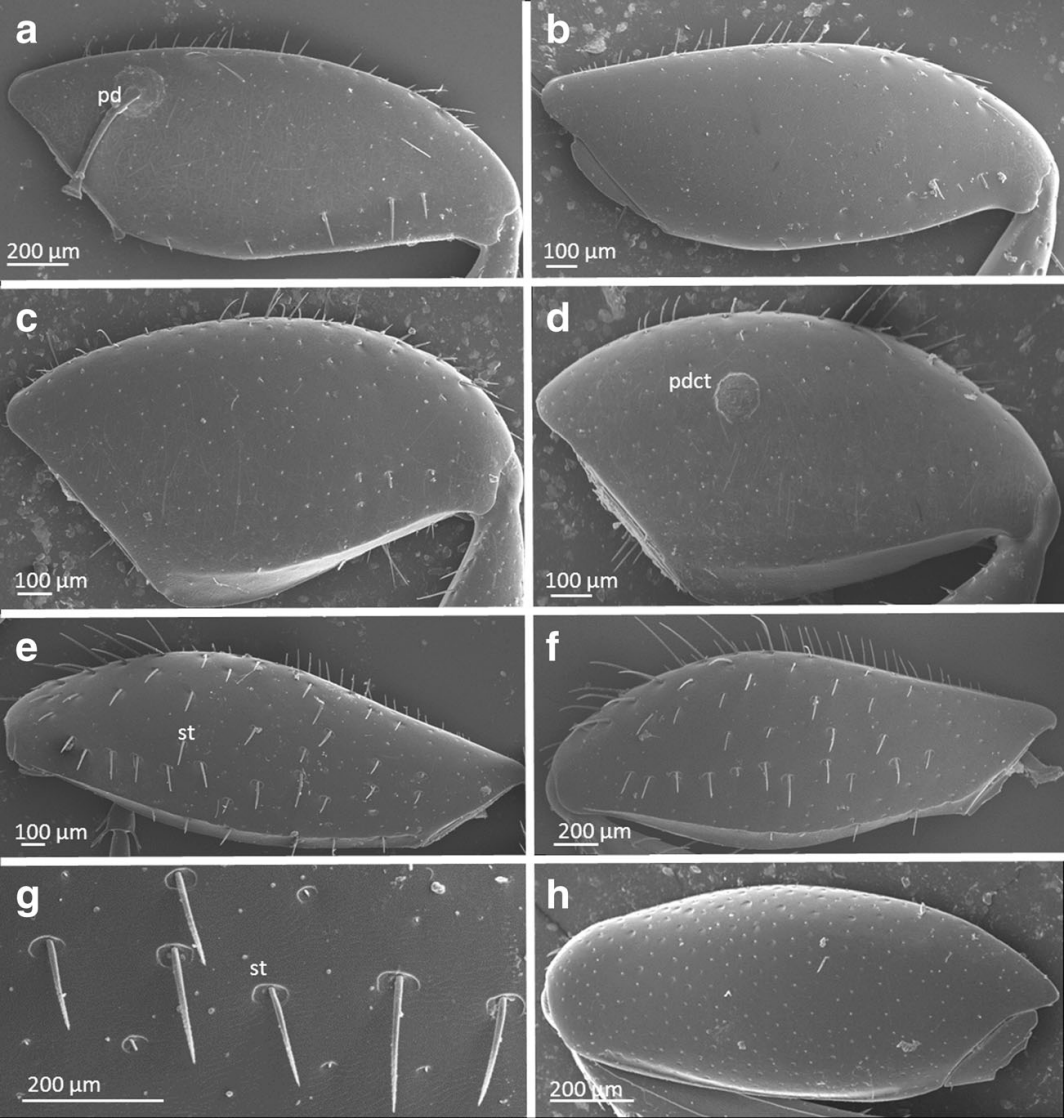
SEM images of ventral side of femora of the third pair of legs in females (**a**) and males (**b**) of *Aphodius prodromus*, females (**c**) and males (**d**) of *Aphodius fimetarius*, females (**e**) and males (**f**, **g**) of *Onthophagus nuchicornis*, **h** *Margarinotus carbonarius. pd* pedicel, *pdct* pedicel carrier terminus, *st* seta

The area of femora in males of *A. prodromus* is larger (7.8 (±0.3) × 10^5^ μm^2^) than in females (5.9 (±0.2) × 10^5^ μm^2^) (ANOVA, *F*_(1,37)_ = 40.675, *P* < 0.0001) (Table 1). The distribution of femoral setae in both sexes is aggregated with most setae located along the femur perimeter. Thus, the main area of femora is smooth (Fig. 3a, b). In both sexes, the setae differ considerably in their length, with many of them being damaged; thus, we did not test whether the differences in length between them are statistically significant. However, females have longer setae (99.1 ± 7.5 μm) when compared with males (74.3 ± 4.1 μm).

The mean surface area in males of *A. fimetarius* is larger (7.0 (±0.2) × 10^5^ μm^2^) than in females (6.6 (±0.2) × 10^5^ μm^2^) ANOVA, *F*_(1,35)_ = 2.677, *P* = 0.111) (Table 1). Similar to *A. prodromus*, the setae are located along the femur perimeter (Fig. 3c, d). Femoral setae are longer in females (46.7 ± 7.9 μm) than in males (33.2 ± 4.5 μm), with many of them being damaged.

Males of *O. nuchicornis* have larger femora (1.3 (±0.05) × 10^6^ μm^2^) than females (1.1 (±0.05) × 10^6^ μm^2^) (ANOVA, *F*_(1,40)_ = 7.915, *P* = 0.008) (Table 1). In both sexes, setae are distributed relatively regularly on the entire femoral surface, with a mean density 3.2 ± 0.1 setae/100,000 μm^2^ in males and 3.5 ± 0.3 setae/100,000 μm^2^ in females (Mann–Whitney *U* test, Z = 0.352, *P* = 0.725) (Fig. 3e–g). Mean seta length for males is 128.5 ± 3.4 and 98.6 ± 2.3 μm for females (ANOVA, *F*_(1,168)_ = 51.233, *P* < 0.00001). Similarly, femoral seta width in males (11.0 ± 0.3 μm) is larger than in females (8.2 ± 0.2 μm) (ANOVA, *F*_(1,161)_ = 79.452, *P* < 0.00001).

The mean surface area of femora in *M. carbonarius* is 5.0 (±0.2) × 10^5^ μm^2^ and is not covered with distinct setae (Fig. 3h) (Table 1). The main part of femoral area in this beetle species has small pits, and every pit has a single seta that does not protrude above the femoral surface.

#### Surface roughness of elytra, propygidium and pygidium

**Fig. 4:**
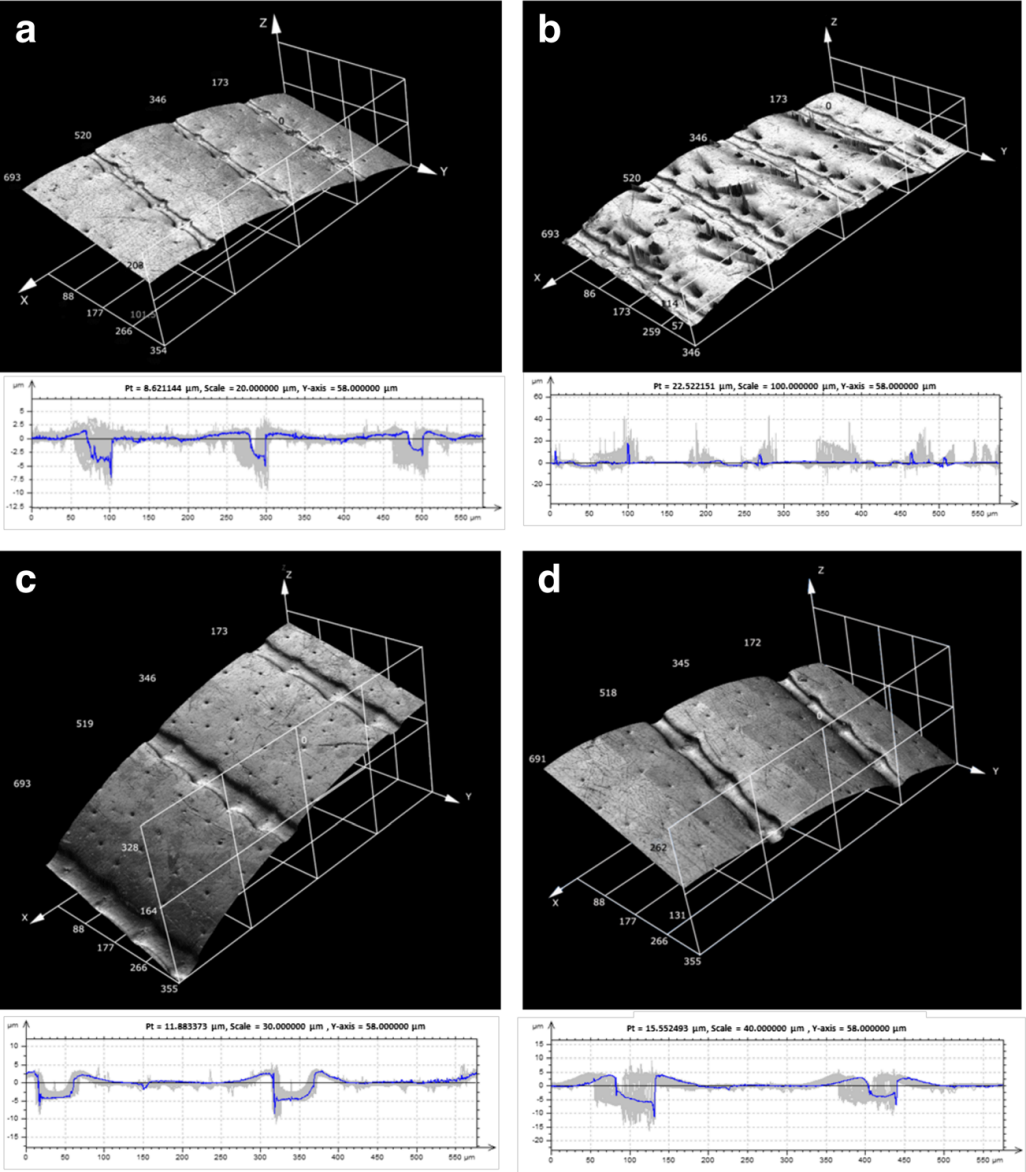
3D surface images and 2D profile images of elytra in females (**a**) and males (**b**) of *Aphodius prodromus*, females (**c**) and males (**d**) of *A. fimetarius. Pt* the amplitude of the displayed profile

**Fig. 5:**
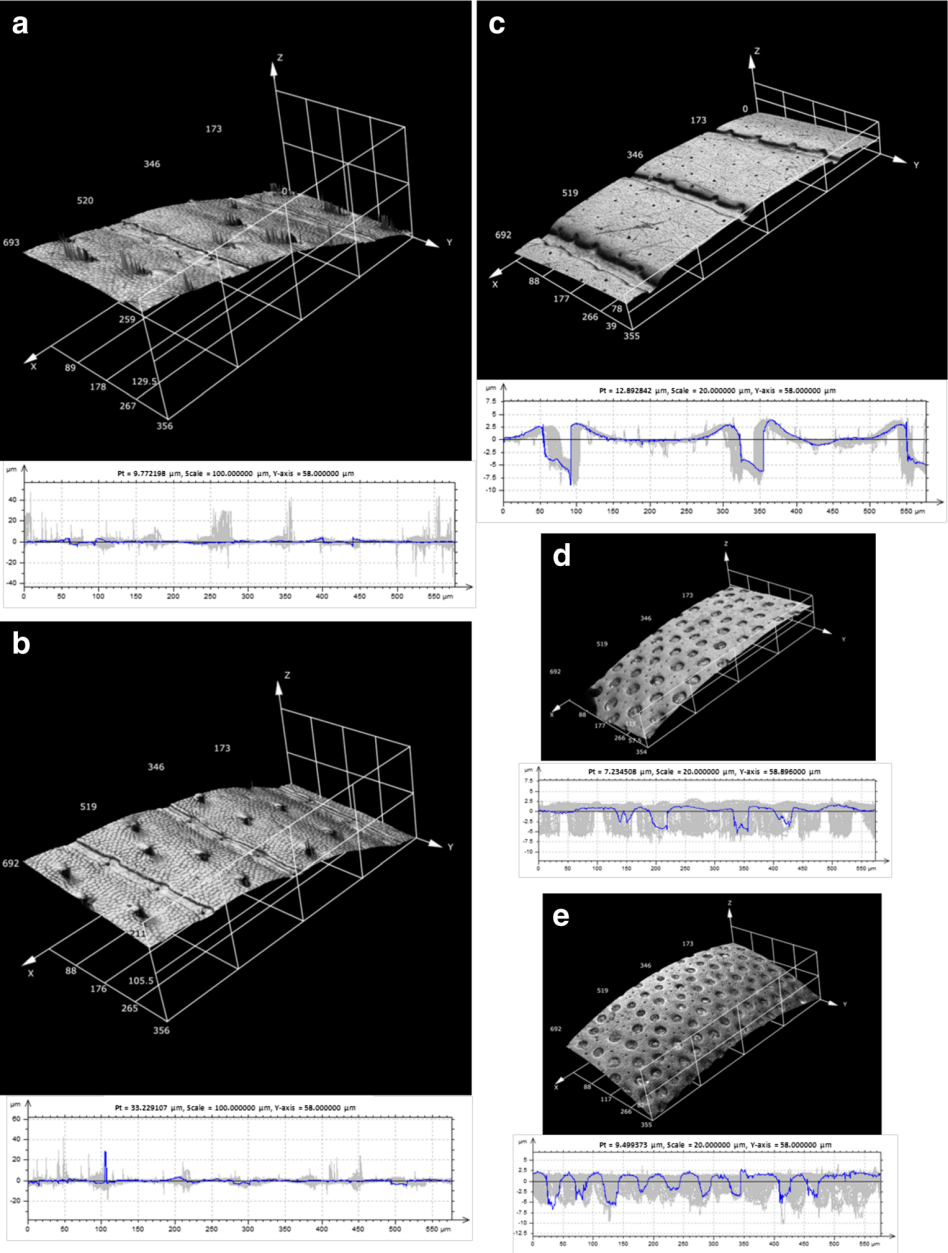
3D surface images and 2D profile images of elytra in females (**a**) and males (**b**) of *O. nuchicornis*, *Margarinotus carbonarius* (**c**), and propygidium (**d**) and pygidium (**e**) in *M. carbonarius. Pt* the amplitude of the displayed profile

Surface texture of studied beetle body parts is presented in Figs. 4 and 5. Values of roughness parameters are presented in Table 4. Data obtained as a result of the analysis of hairy surfaces should be interpreted cautiously because we took into consideration the presence of setae. As mentioned above, setae can be arranged in various ways, with many of them being damaged affecting the CLSM measurements. Nevertheless, the presented results reflect better real surface conditions that the deutonymph meets. The highest values of *Ra* parameter were found for elytra, propygidium and pygidium in *M. carbonarius* and for elytra in females of *A. fimetarius* (Table 4). The maximum height of the profile (*Rz*) turned out to be the highest for elytra in males of *A. prodromus*, females of *O. nuchicornis*, *M. carbonarius* and females of *A. fimetarius*. The lowest values of *Rv* parameter were found for elytra in males of *A. fimetarius* and females of *O. nuchicornis* (Table 4).

**Table 4:** Surface profile characteristics of beetle elytra, propygidium and pygidium

Beetle species/sex	*Ra* (μm)	*Rz* (μm)	*Rv* (μm)	*Sa* (μm)	*Sz* (μm)	*Sv* (μm)
	Mean ± SD	Mean ± SD	Mean ± SD			
	*N* = 138	*N* = 138	*N* = 138	*N =* 1	*N =* 1	*N =* 1
*Aphodius prodromus* F (ely)	0.69 ± 0.16	7.25 ± 1.36	5.29 ± 1.07	0.79	37.59	10.12
*A. prodromus* M (ely)	0.90 ± 0.24	15.73 ± 8.18	4.28 ± 1.59	1.13	63.96	20.48
*Aphodius fimetarius* F (ely)	1.05 ± 0.12	10.30 ± 1.55	6.92 ± 1.33	1.07	17.44	11.56
*A. fimetarius* M (ely)	0.76 ± 0.13	6.80 ± 3.93	2.99 ± 3.24	1.45	32.86	17.40
*Onthophagus nuchicornis* F (ely)	0.63 ± 0.17	12.53 ± 10.45	3.76 ± 2.95	0.86	101.25	49.35
*O. nuchicornis* M (ely)	0.80 ± 0.18	7.44 ± 3.30	4.15 ± 1.38	0.94	85.75	37.69
*Margarinotus carbonarius* (ely)	1.48 ± 0.08	11.27 ± 0.56	7.46 ± 0.55	1.57	15.31	9.73
*M. carbonarius* (pro)	1.41 ± 0.55	7.40 ± 1.57	5.19 ± 1.13	1.52	13.02	8.76
*M. carbonarius* (pyg)	1.01 ± 0.43	5.94 ± 1.70	4.27 ± 1.33	1.16	15.16	11.33

*F* females, *M* males, *ely* elytra, *pro* propygidium, *pyg* pygidium, *Ra/Sa* arithmetical mean deviation of the assessed profile/surface, *Rz*/*Sz* maximum height of profile/surface, *Rv*/*Sv* maximum profile/surface valley depth

#### The influence of the presence of setae on deutonymph attachment

**Fig. 6:**
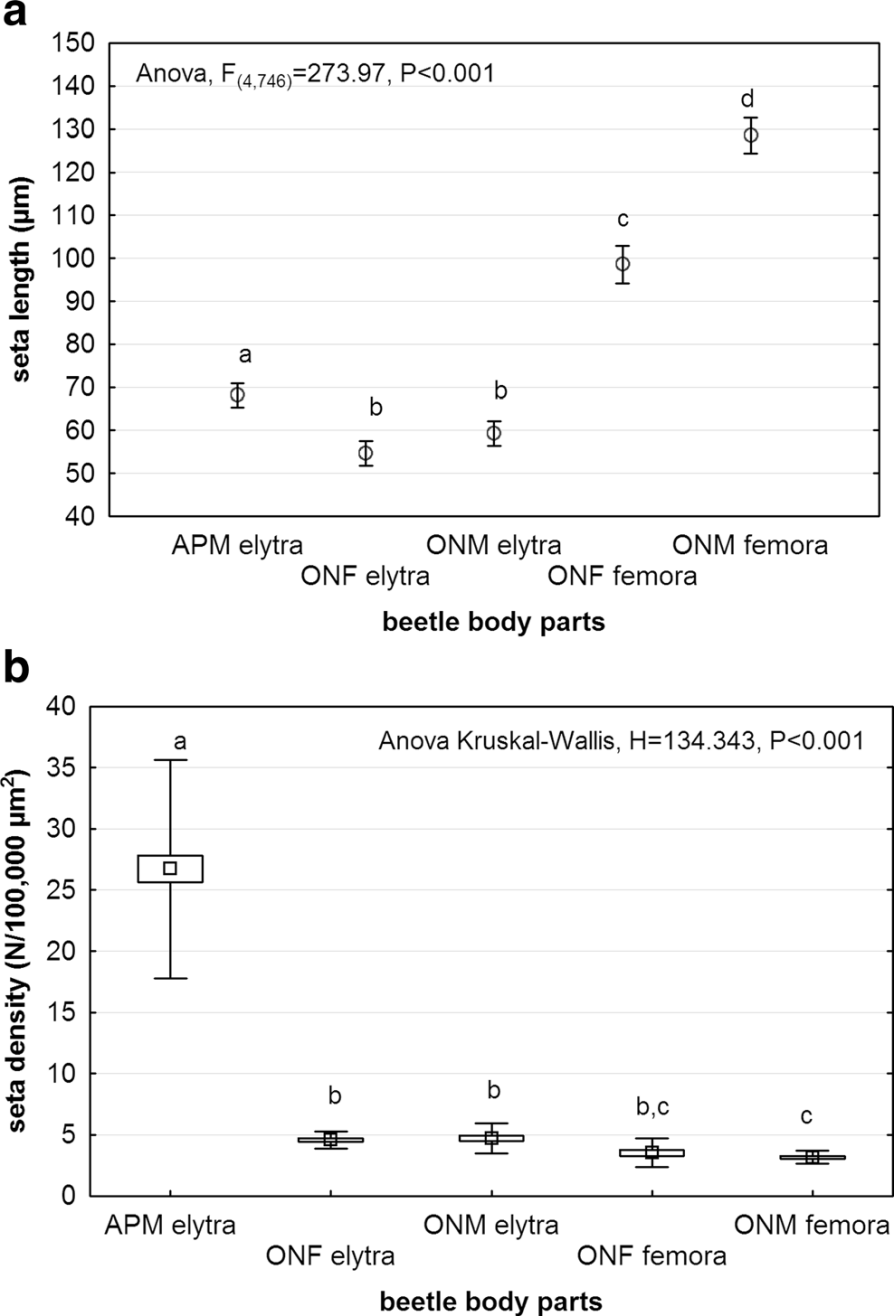
Length (**a**) and density (**b**) of elytral and femoral setae. *APM* males of *Aphodius prodromus*, *ONF* females of *Onthophagus nuchicornis*, *ONM* males of *O. nuchicornis. Markers and vertical bars* in **a** denote mean and confidence intervals, whereas *boxes and vertical bars* in **b** denote standard error of the mean and standard deviation, respectively. *Different letters* denote significant differences between experimental groups in pairwise comparisons (*P* < 0.05)

Numbers of beetle individuals collected and infested with deutonymphs are presented in Table 5. Differences in seta length and seta density between beetle species are shown in the Fig. 6. The analysis revealed that of the tested setae, the femoral setae in males of *O. nuchicornis* were the longest and the elytral setae in males and females of this species were the shortest (ANOVA, *F*_(4,746)_ = 273.97, *P* < 0.0001) (Fig. 6a). No statistically significant differences were found between lengths of elytral setae in both sexes of *O. nuchicornis*. The highest seta density was found for elytral setae in males of *A. prodromus* and the lowest for femoral setae in both sexes of *O. nuchicornis* (ANOVA Kruskal-Wallis, *H* = 134.343, *P* < 0.0001) (Fig. 6b).

**Table 5:** Beetle species analysed with the information on number of beetles collected (*N*), number of beetles infested (*k*), number of beetles with infested elytra (*kE*) and number of beetles with infested femora (*kF*)

Beetle species		*N*	*k*	*kE*	*kF*
*Aphodius prodromus*	F	2,909	819	335	430
	M	2,172	668	132	454
*Aphodius fimetarius*	F	316	180	88	147
	M	280	169	98	138
*Onthophagus nuchicornis*	F	766	467	18	430
	M	782	516	27	464
*Margarinotus carbonarius*		505	245	211^a^	112

*F* females, *M* males

^a^The number includes individuals with infested propygidium and pygidium

**Fig. 7:**
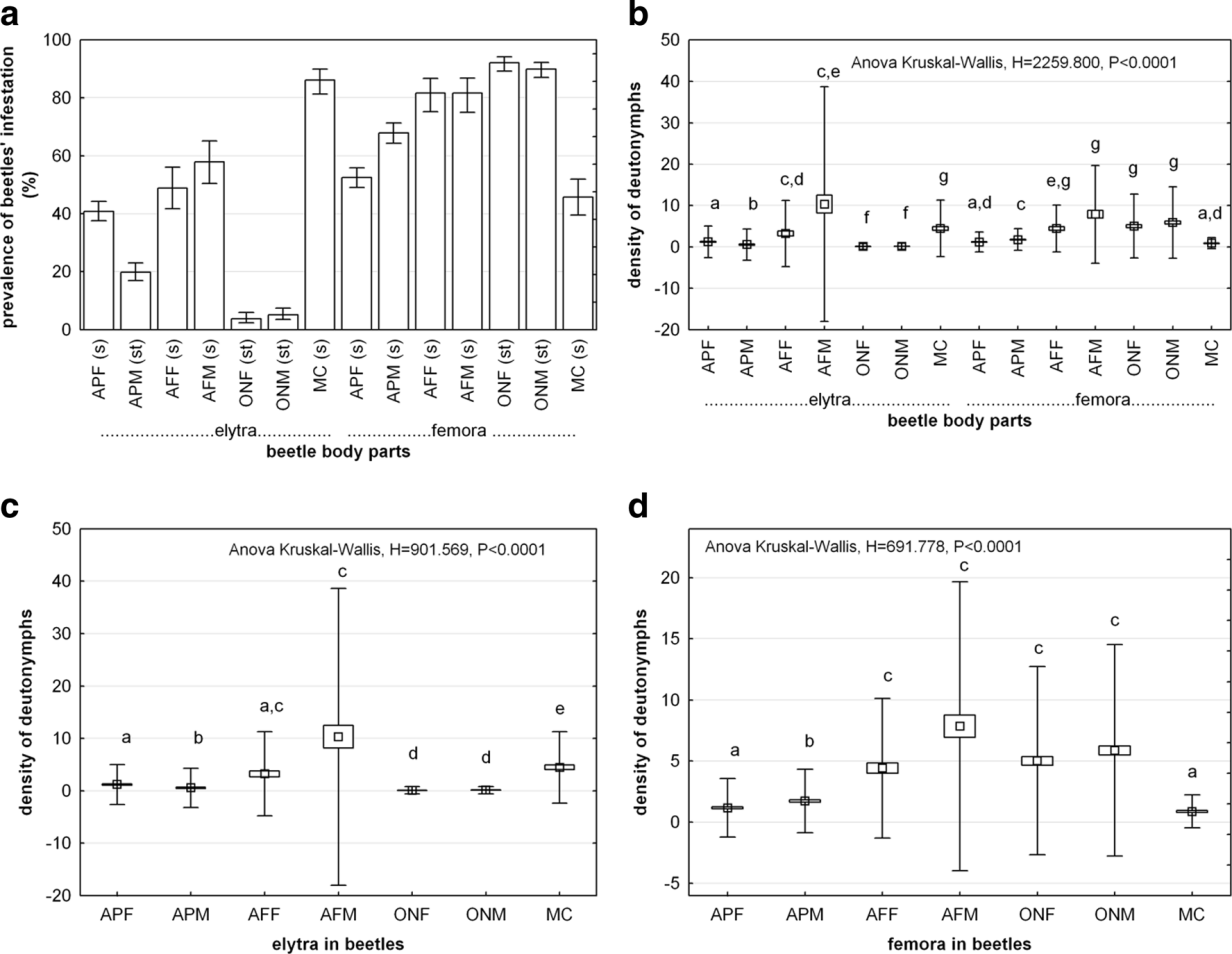
Incidence of infestation of *Uropoda orbicularis* on elytra and femora of the third pair of legs in studied beetle species. **a** Prevalence of beetle infestation with deutonymphs attached to elytra and femora (*vertical bars* denote 95 % confidence intervals), **b** density of deutonymphs on elytra and femora, **c** density of deutonymphs on elytra, **d** density of deutonymphs on femora. *s* smooth body part, *st* body part covered with setae, *APF* females of *Aphodius prodromus*, *APM* males of *A. prodromus*, *AFF* females of *Aphodius fimetarius*, *AFM* males of *A. fimetarius*, *ONF* females of *Onthophagus nuchicornis*, *ONM* males of *O. nuchicornis*, *MC Margarinotus carbonarius. Boxes and vertical bars* denote standard error of the mean and standard deviation, respectively. *Different letters* denote significant differences between experimental groups in pairwise comparisons (*P* < 0.05)

The highest values for the prevalence of beetles and the density of deutonymphs were found for smooth body parts or these covered with setae of low density (Fig. 7). The correlation between seta and deutonymph densities was weak, negative (*r* = −0.3) and not statistically significant (*P* = 0.270). The highest prevalence of beetles with deutonymphs was recorded for femora in *O. nuchicornis*, elytra in *M. carbonarius* (including propygidium and pygidium) and femora in *A. fimetarius* (Fig. 7a). When comparing deutonymph densities between beetles, it turned out that most heavily infested were elytra and femora in males of *A. fimetarius*, femora in both sexes of *O. nuchicornis*, elytra in *M. carbonarius* and femora in females of *A. fimetarius* (ANOVA Kruskal-Wallis, *H* = 2259.800, *P* < 0.0001) (Fig. 7b). No statistically significant differences were found in deutonymph densities on elytra and femora between both sexes in *A. fimetarius* and *O. nuchicornis* (Fig. 7b–d). The comparison of deutonymph densities between elytra only revealed the lowest values of infestation for both sexes of *O. nuchicornis* and males of *A. prodromus* (ANOVA Kruskal-Wallis, *H* = 901.569, *P* < 0.0001) (Fig. 7c). Surprisingly, elytra in males of *A. prodromus* were more heavily infested, although seta density and length were higher than these in both sexes in *O. nuchicornis*. In case of femora, the highest values of deutonymph densities were found for both sexes of *A. fimetarius* and *O. nuchicornis* (ANOVA Kruskal-Wallis, *H* = 691.778, *P* < 0.0001) (Fig. 7d).

#### The influence of surface sculpture on deutonymph attachment

**Fig. 8:**
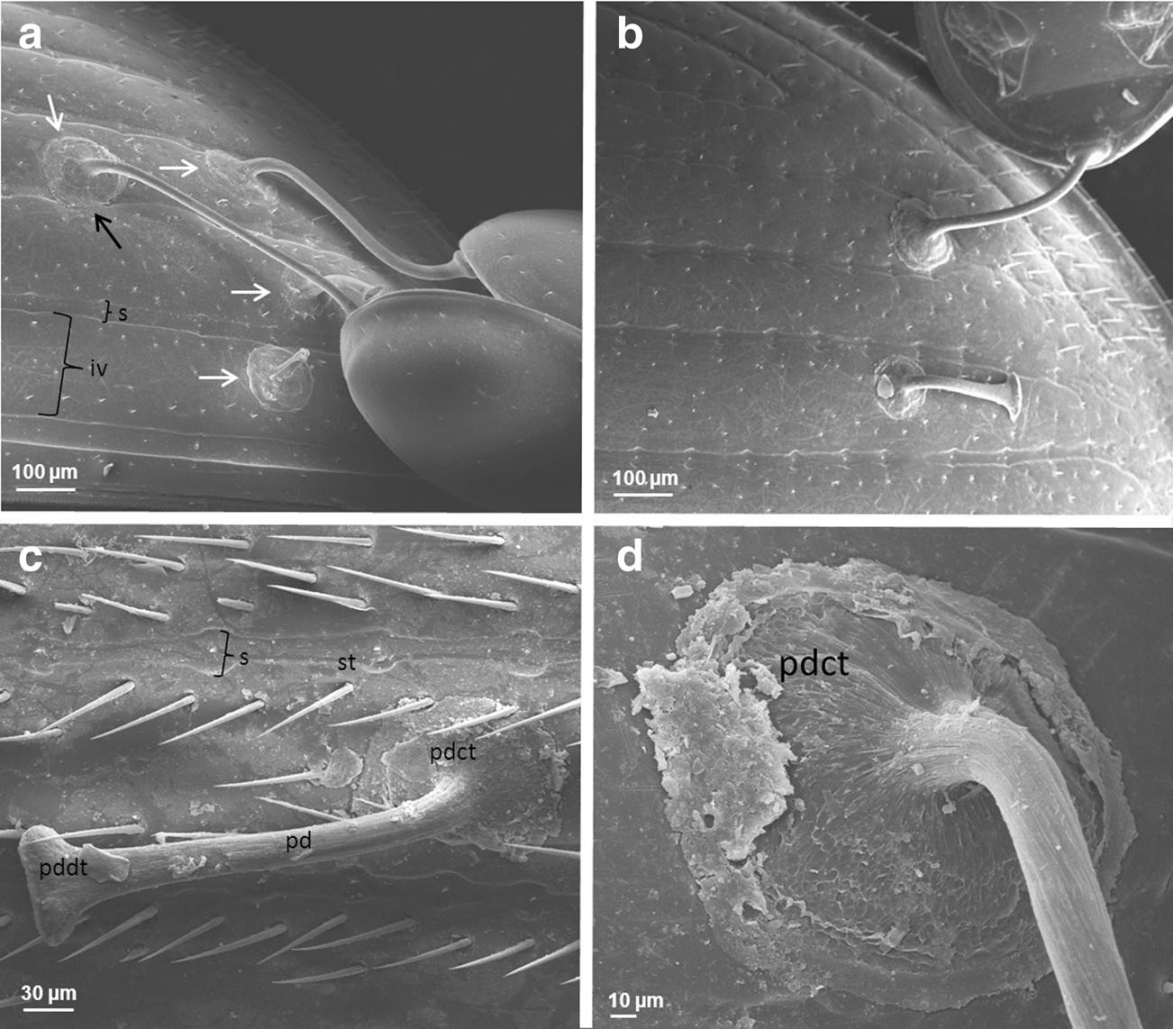
Localization of pedicels of *Uropoda orbicularis* within elytra of females and males of *Aphodius prodromus*. **a**, **b** Pedicels attached to elytra in females; **c** a pedicel attached to interval in male; **d** pedicel carrier terminus attached to interval. *White arrows* indicate pedicel carrier terminus; *black arrow* indicates a part of pedicel carrier terminus attached partially to stria. *iv* interval, *s* stria, *st* seta, *pd* pedicel, *pdct* pedicel carrier terminus, *pddt* pedicel deutonymph terminus

**Fig. 9:**
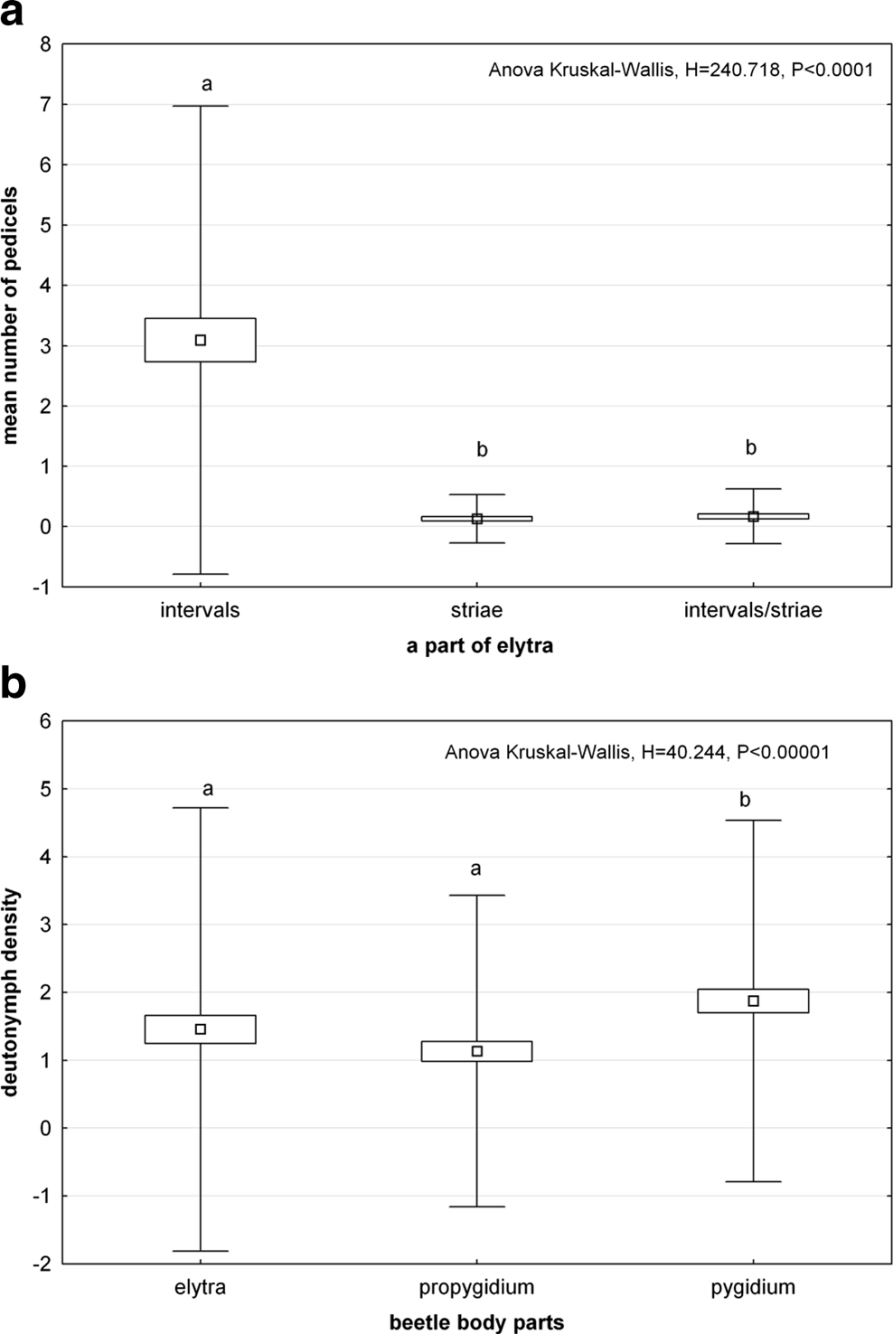
Mean number of pedicels of *Uropoda orbicularis* attached to elytral intervals and striae of *Aphodius prodromus* (*N* = 60) and *A. fimetarius* (*N* = 60) (**a**) and density of phoretic deutonymphs on elytra, propygidium and pygidium in *Margarinotus carbonarius* (**b**). *Boxes and vertical bars* denote standard error of the mean and standard deviation, respectively. *Different letters* denote significant differences between experimental groups in pairwise comparisons (*P* < 0.05)

**Fig. 10:**
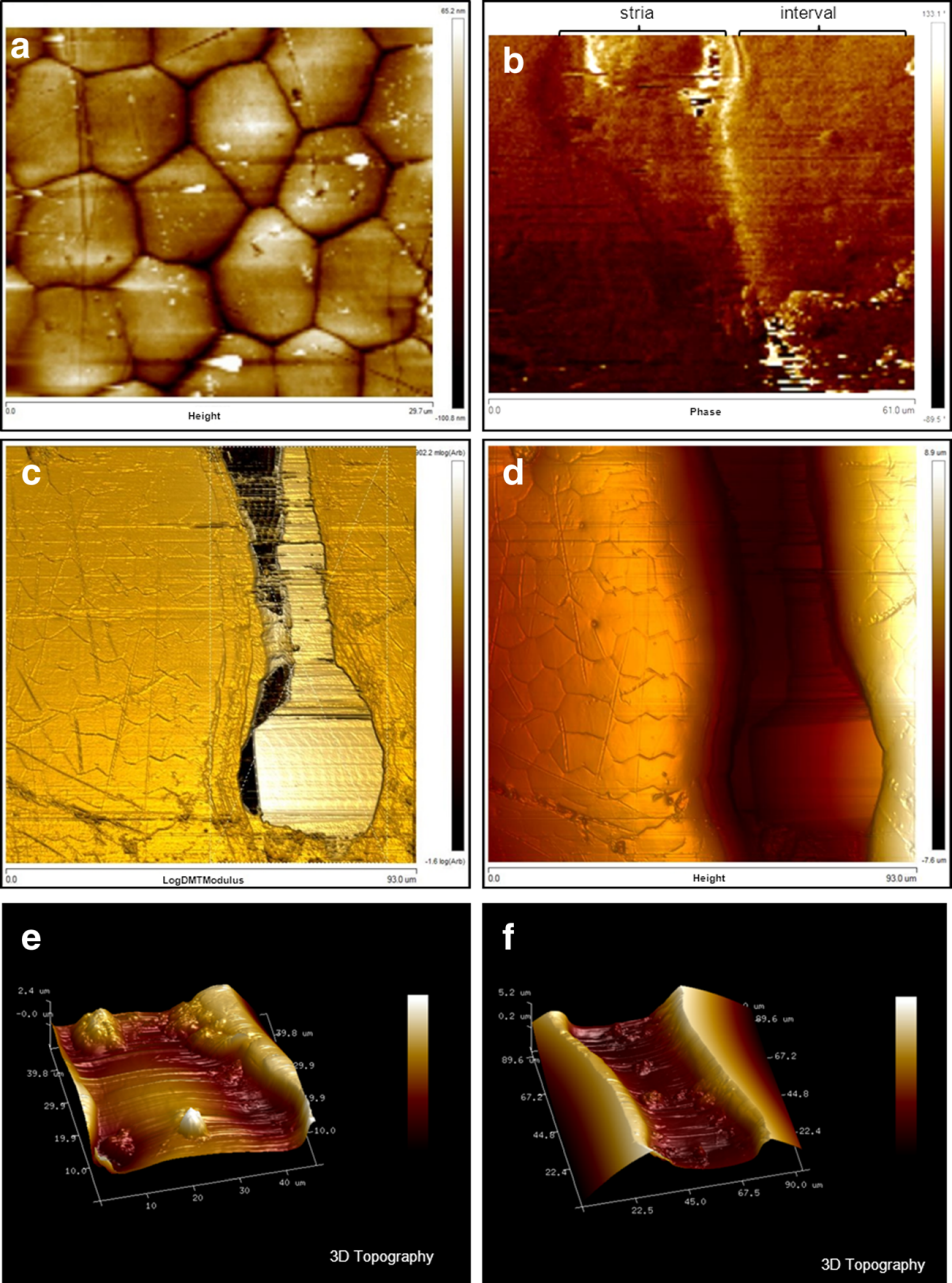
AFM images of the elytral interval and stria in males of *Aphodius fimetarius*. **a** Height of the polygonal surface of the interval, **b** phase, **c** logDMTModulus, **d** height, **e** 3D topography of the stria surface, **f** 3D topography of the stria

The study revealed that within elytra, deutonymphs may attach to (i) intervals, (ii) partially to intervals and striae and (iii) striae (Fig. 8). The differentiation was made according to the location of the centre of the pedicel carrier terminus. In the first case, the entire pedicel carrier terminus, which according to our observation has a mean diameter of 116.8 ± 3.3 μm (*N* = 38), was located within the interval. In the second case, the central part of pedicel carrier terminus was located within the interval but reached partially the striae. In the last case, the central part of pedicel carrier terminus was located within the stria but reached intervals on both sides of the stria. Pedicels were attached to intervals in most individuals of *Aphodius fimetarius* (96.7 %) and *Aphodius prodromus* (93.3 %). The analysis conducted on data pooled from two species showed that differences in number of pedicels attached to the analysed areas of elytra were statistically significant (ANOVA Kruskal-Wallis, *H* = 240.718, *P* < 0.0001) (Fig. 9a). Among 406 analysed pedicels, 371 pedicels (91.4 %; CI 88.3–93.8) were attached to intervals. Only 15 pedicels (3.7 %, CI 2.3–6.0) had the central part of their carrier terminus located directly in the stria and 20 pedicels (4.9 %; CI 3.2–7.5) were attached partially to intervals and striae. The AFM measurements showed that intervals and striae in males of *A. fimetarius* differed in topography and logDMT modulus, whereas no difference was found in the phase between these two elytral areas (Fig. 10). Interval surface is the highest along its both sides at the border with the stria. Surface of elytral interval turned out to be more elastic than the surface of the striae.

**Fig. 11:**
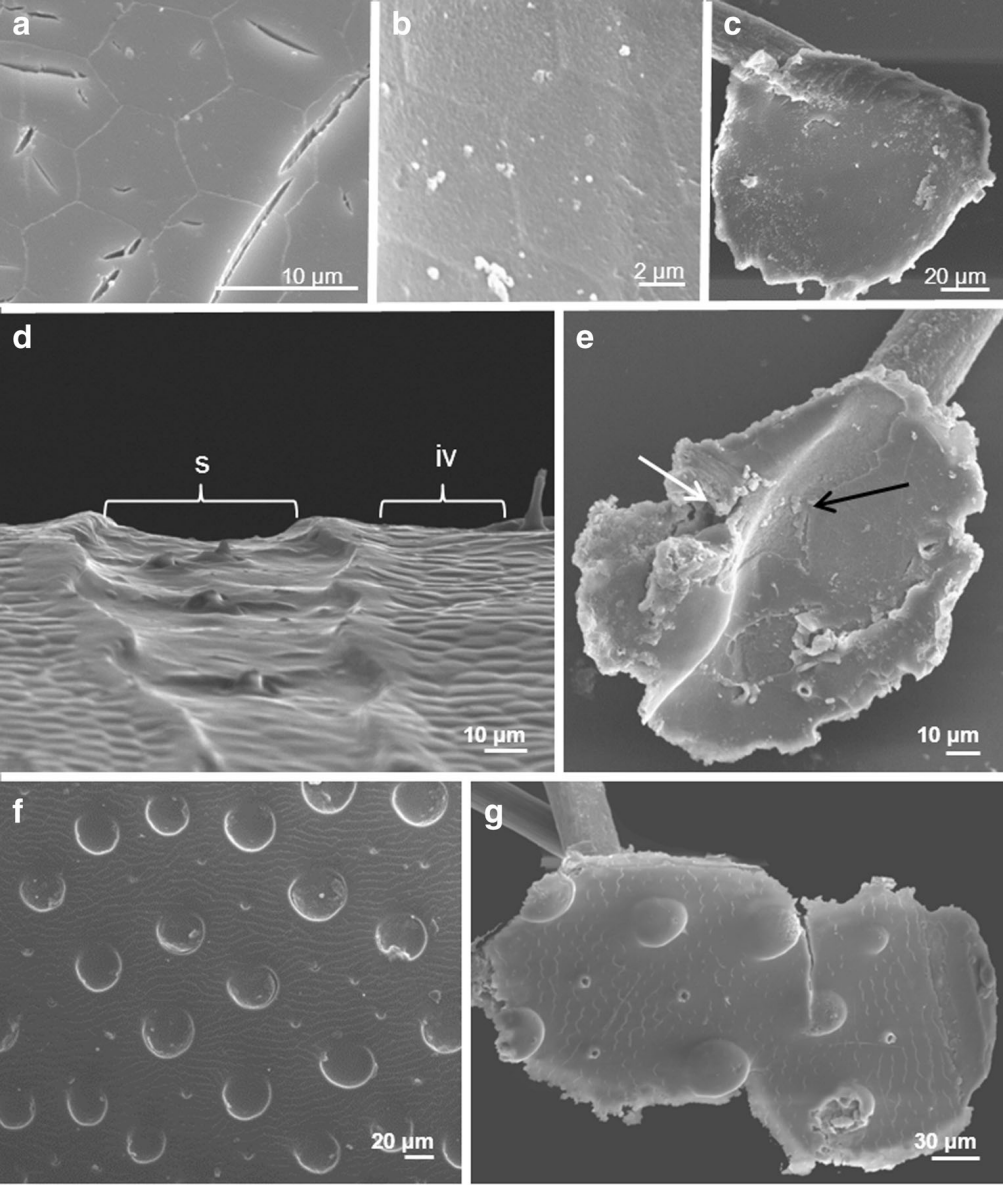
SEM images of the attachment surfaces and bottom side of pedicels’ carrier terminus attached to these surfaces. **a** Polygonal surface texture of elytral interval in *Aphodius fimetarius*, **b**, **c** bottom side of the carrier terminus of the pedicel attached to interval in males of *A. fimetarius* (**b** at high magnification), **d** a stria and an interval in males of *A. fimetarius*, **e** bottom side of pedicel carrier terminus attached partially to elytral stria and interval in males of *A. fimetarius*, **f** surface of pygidium in *Margarinotus carbonarius*, **g** bottom side of carrier pedicel terminus attached to pygidium in *M. carbonarius. s* stria, *iv* interval; the *white arrow* indicates a depression that corresponds to the elytral interval, and the *black arrow* indicates longitudinal convexity that corresponds to the elytral stria

The analysis of deutonymph densities on propygidium and pygidium of *M. carbonarius* showed that deutonymphs may attach to surfaces densely punctuated (Fig. 9b). When comparing particular dorsal body parts separately (elytra, propygidium and pygidium), the highest deutonymph density was found for pygidium and the lowest for propygidium (ANOVA Kruskal-Wallis, *H* = 40.244, *P* < 0.00001) (Fig. 9b). The SEM study showed that the bottom side of the pedicel carrier terminus matches precisely to the surface profile (Fig. 11). Even gaps between small irregularities were filled with the pedicellar secretion (Fig. 11a, b).

## Discussion

A huge variety of animal attachment devices, their structure, biomechanical properties and biological function were intensively studied (Gorb 2001; Persson and Gorb 2003; Gorb and Varenberg 2007; Gorb 2008; Gorb and Gorb 2009; Wolff and Gorb 2012). Among them, attachment strategies involving wet adhesion turned out to be of technical and biomedical importance (Allmeling et al. 2008; Haller et al. 2011; Scheibel 2012). Pedicel of Uropodina mites is an example of biological glue, but when comparing with other sticky secretions produced by animals, e.g., byssus threads or spider silk, it received little attention. One of the basic aspects of studies on the mechanism of attachment in animals is the analysis of surface texture and mechanical properties of attachment surfaces (Huber et al. 2007; Santos et al. 2008; Gorb and Gorb 2009; Wolff and Gorb 2012). Although morphology, topography and mechanical properties of beetle elytra were studied in detail (Sun and Bhushan 2012; Sun et al. 2012), they were never analysed in context of attachment surfaces for phoretic deutonymphs of Uropodina. Faasch (1967) observed phoretic behaviour in two Uropodina species—*Uropoda orbicularis* and *Uroobovella marginata* (C. L. Koch, 1839) revealing that deutonymphs may attach not only to smooth surfaces of their carriers, but also to the plastic and glass walls of rearing containers. In turn, Mertins and Hartdegen (2003) studied deutonymphs of *Fuscuropoda marginata* (= *Uroobovella marginata*) attached to the head, right forelimb and the basolateral tail of a small lizard—*Scincella lateralis* (Say in James, 1823), whereas Rives and Barnes (1988) observed deutonymphs of the abovementioned mite species attached to the legs of broiler chicks.

The presented results show that deutonymphs may attach to surfaces of various morphology and topography. We observed deutonymphs attached to the following kinds of surfaces: (i) smooth (without setae or with microseta cover), (ii) hairy, i.e., covered with distinct setae of various densities and lengths, (iii) flat and (iv) sculptured. Taking into consideration the influence of setae on deutonymph attachment, it turned out that smooth surfaces and surfaces with low seta density were most intensively occupied by mites. Some surfaces turned out to be covered with microsetae. They were so short that they did not protrude above the carrier body surface; hence, such surfaces looked as if they were not covered with setae. The relation between deutonymph burden and the presence of setae was particularly well seen in case of females and males of *Aphodius prodromus*. Individuals of both sexes were equally numerous in the studied community occurring at the same time, and therefore were available for mites with the same probability. The presence of elytral setae in males may explain lower infestation with phoretic deutonymphs. Similarly, a low infestation with mites was recorded for seta-covered elytra in females and males of *O. nuchicornis*. Therefore, it can be concluded that the presence of setae makes somehow the pedicel attachment difficult. These results raise a question of the amount of space required for deutonymph attachment. According to our measurements, the pedicel carrier terminus in *U. orbicularis* has a mean diameter of 116.8 ± 3.3 μm, and the smallest mean distance between setae (100.5 ± 3.2 μm) was recorded just for elytral setae in males of *A. prodromus*. Moreover, these setae lie flat along the intervals, which makes that they cover most of elytral surface. Probably, the small distance between the setae, as well as their arrangement, is the cause of low deutonymph infestation. On the contrary, the mean distance between elytral setae in females and males of *O. nuchicornis* is more than the mean diameter of pedicel carrier terminus. In addition, elytral setae in males and females of *O. nuchicornis* are shorter than elytral setae in males of *A. prodromus*; hence, they do not cover the entire elytral surface. It means that phoretic deutonymphs had more space for the attachment when they climb on individuals of *O. nuchicornis*. Therefore, one should expect that deutonymph density on elytra in *O. nuchicornis* will be higher than deutonymph density on elytra in males of *A. prodromus* because they provide a better attachment site. It turned out, however, that elytra in both sexes of *O. nuchicornis* were characterised with lower deutonymph infestation. This fact suggests that factors other than morphology of the carrier surface may affect pedicel attachment. Representatives of *Aphodius* and *Onthophagus* differ significantly in their reproductive behaviour (Hanski and Cambefort 1991). Females of aphodiid beetles lay their eggs into the dung, upper layer of soil or between soil and dung and do not show parental care. In *Onthophagus* beetles, both sexes participate in tunnel digging, and females stay in breeding cells to protect their progeny. Therefore, relatively small number of deutonymphs attached to elytra in individuals of *O. nuchicornis* may be related to tunnel digging. Pedicels of deutonymphs attached to more exposed body parts such as the upper part of elytra could be simply cut off when tunnel digging. This explains why more deutonymphs were found on legs in this beetle species. Although femora are covered with long setae, their density and the distance between them are low enough to not disturb deutonymph attachment. A similar pattern of Uropodina deutonymph infestation was observed for burying beetles of the genus *Nicrophorus* (Silphidae), which burry carcasses of small carrion in soil (Schwarz et al. 1998). To sum up, it can be concluded that morphology of the carrier surfaces is a vital factor affecting deutonymph attachment but cannot fully explain the observed results.

The attachment ability to rough surfaces in biological systems were the subject of many studies (Persson and Gorb 2003; Huber et al. 2007; Santos et al. 2008; Wolff and Gorb 2012; Ditsche et al. 2014; Spinner et al. 2014). The height of irregularities is the main factors affecting adhesion (Gorb 2010). In general, high surface roughness promotes adhesion, but strong adhesion will also occur between two ideally smooth substrates. Such adhesion may involve van der Waals forces and hydrogen bonds and will occur if sufficient contact area at the interface between the substrate and the adhesive is reached (Gorb 2008). In our study, we considered deutonymph attachment to surfaces of two kinds: elytra with long striae and strongly punctuated propygidium and pygidium. Bajerlein and Witaliński (2014) gave the general information that deutonymphs of *U. orbicularis* attach frequently to the intervals of *A. prodromus* elytra. In the present article, we support this information with detailed statistical analysis conducted for pedicels of *U. orbicularis* collected from different aphodiids: *A. prodromus* and *A. fimetarius*. The analysis of deutonymph localization on elytra of these beetles has clearly shown that deutonymphs prefer flat surfaces of intervals. A high number of pedicels attached to intervals is striking suggesting that phoretic deutonymphs are able to recognise whether the surface is suitable for their attachment or not. It is, however, difficult to explain the way in which they do so. It can be assumed that the deutonymphal anal setae, e.g., setae situated near the anus, can be involved somehow in the process of surface recognition. Secretion and formation of the pedicel has been described by Faasch (1967). This author has observed that phoretic deutonymphs, after reaching a carrier body surface, start searching for an appropriate place for attachment. The process of pedicel formation starts when the deutonymph sits with its anus on the carrier’s body and then it secretes a certain amount of pedicellar substance to attach itself. Therefore, the deutonymph must get physically in contact with the attachment surface while forming the pedicel. The first secreted portion of the pedicellar substance makes a carrier terminus. The finding of most deutonymphs attached to intervals could be simply explained by the fact that elytral intervals make most of the elytral surface. Therefore, the probability of attaching to interval will be higher than attaching to the striae. Nevertheless, the accuracy in attaching to intervals is striking and could not just be explained by coincidence. Different microscopic techniques used in our studies (SEM, CLSM, AFM) showed that the interval surface is the highest along its both sides, at the border with the striae. It can be assumed that the deutonymph recognises this bulge in surface profile which makes attachment difficult or impossible. The mean width of anus in deutonymphs of *U. orbicularis* is on average 70 μm (personal observations, measurements from SEM digital images); hence, it is wider than the mean width of striae in females and males of *A. prodromus* and *A. fimetarius*. Probably the anus stops on these irregularities and as a result does not allow to get the mite in a proper physical contact with the stria surface. In this case, elytral surface topography seems to be the most important factor affecting pedicel attachment and not the other studied by us using AFM properties. According to our results, intervals and striae did not show differences in phase, hence the adhesion was similar. Although intervals turned out to be more elastic than striae, probably the differences in log DMT modulus were so small that they did not affect pedicel attachment.

The results on deutonymph infestation on elytra in aphodiids are in contrary to results obtained from the study of deutonymph density on propygidium and pygidium in *M. carbonarius*. Densely punctuated propygidium and pygidium were intensively infested with phoretic deutonymphs. This may be due to the fact that values of mean surface roughness for pygidium and propygidium are the ones of the highest among all studied, but, on the contrary, values of maximum height profile are relatively low. This means that surfaces of propygidium and pygidium are rough enough to affect effective bonding, but the height of irregularities was not so high to disturb mite attachment. Previous results on topical specificity of *U. orbicularis* deutonymph revealed that most deutonymphs attached to elytra are located within their posterior parts, i.e., within the elytral slope and on the apex (Bajerlein and Błoszyk 2004). It was hypothesised that such location would preserve phoretic deutonymphs from being detached while carrier digs in the soil. Propygidium and pygidium in *M. carbonarius* correspond in their location to the apex of elytra in aphodiid species, which may explain why these two segments were often occupied by mites. Following this hypothesis, it can be assumed that deutonymphs would attach to pygidium and propygidium, regardless of their topography. This implies that although irregular surfaces of propygidium and pygidium may positively affect the adhesion throughout the increase in contact area, they are not crucial for the effective bonding between the pedicellar substance and the carrier body surface.

To our knowledge, the pedicel has never been the subject of biomechanical studies. It seems that it possesses both elastic and viscous properties. Photographs obtained using SEM showed that the contact surface of the pedicel carrier terminus matches the substratum profile. It means that the adhesive substance fills gaps even between small surface irregularities. This makes the close proximity between two substrata possible, which is required to achieve strong adhesion. The attachment force depends directly on the area of real contact, which was previously shown for tube feet of echinoderms (Santos et al. 2008).

In this article, we present the first detailed analysis of morphology and topography of attachment surfaces for phoretic deutonymphs of Uropodina mites using various microscopic techniques. The results confirmed earlier observations that deutonymphs prefer smooth surfaces. However, it turned out that surfaces covered even with long setae but of low density may be selected for attachment. The next step in explaining the revealed relation between carrier surface morphology/topography and deutonymph attachment should involve experiments that will verify the adhesive strength of the pedicel in relation to various substratum profiles. This will not only lead to better understanding of the mechanism of pedicel attachment but will also allow for the better understanding of the evolution of the phenomenon of phoresy in this mite group.
